# Effect of SF and GGBS on Pore Structure and Transport Properties of Concrete

**DOI:** 10.3390/ma17061365

**Published:** 2024-03-16

**Authors:** Wei Chen, Mengmeng Wu, Yue Liang

**Affiliations:** School of Civil Engineering, Architecture and Environment, Hubei University of Technology, Wuhan 430068, China; chenwei@hbut.edu.cn (W.C.); 102110987@hbut.edu.cn (M.W.)

**Keywords:** silica fume, ground granulated blast-furnace slag, transport properties, pore structure, NMR, MIP

## Abstract

Ground Granulated Blast-Furnace Slag (GGBS) and silica fume (SF) are frequently utilized in gel materials to produce environmentally sustainable concrete. The blend of the two components contributes to an enhancement in the pore structure, which, in turn, increases the mechanical strength of the material and the compactness of the pore structure and decreases the permeability, thereby improving the durability of the concrete. In this study, the pore structures of GGBS and SF blends are assessed using Nuclear Magnetic Resonance (NMR) and Mercury Intrusion Porosimetry (MIP) tests. These methodologies provide a comprehensive evaluation of the effect of GGBS and SF on the pore structure of cementitious materials. Results showed that the addition of SF and GGBS reduces the amount of micro-capillary pores (10 < d < 100 nm) and the total pore volume. The results indicate that the transport properties are related to the pore structure. The incorporation of SF reduced the permeability of the concrete by an order of magnitude. The pore distribution and pore composition had a significant effect on the gas permeability. The difference in porosity obtained using the MIP and NMR tests was large due to differences in testing techniques.

## 1. Introduction

High-performance concrete (HPC) is characterized by its high strength, high modulus of elasticity, high density, and low permeability. This typical porous material has varied pore shapes, a complex pore structure, and a broad range of pore size distribution spanning microscopic, mesoscopic, and macroscopic scales [1,2,3]. Mineral admixtures are incorporated into concrete to modify its pore structure, exerting influence on its strength, chloride diffusivity, permeability and other performance characteristics [4,5,6]. The major mineral admixtures used in concrete include silica fume (SF), Ground Granulated Blast-Furnace Slag (GGBS), fly ash (FA), metakaolin, and rice husk ash [7,8,9].

Previous studies have showed that the GGBS can densify the microstructure [10,11], reduce permeability and the heat of hydration [12,13,14], and improve long-term strength [14]. Microstructural analysis of GGBS in concrete using Scanning Electron Microscopy (SEM) revealed that the addition of GGBS produces a denser matrix populated with more angular particles, resulting in improved bonding properties compared to plain concrete, which contains more spherical particles leading to suboptimal bonding properties. These SEM results are corroborated by Energy Dispersive Spectroscopy (EDS) findings, which demonstrated that, compared to normal concrete, the concrete containing GGBS exhibited higher levels of Ca, Si, and Al. This elevated content contributes to the increased formation of both calcium silicate hydrate (C-S-H) gel and calcium aluminate silicate hydrate (C-A-S-H), leading to improved strength [15]. Singh, P. et al. [16] also found that, regardless of the addition of hydrogen, the addition of GGBS improved the distribution of particles in the pulp and increased the fluidity; meanwhile, moderate use of GGBS enhanced the quality of concrete. However, the compressive strength and durability of concrete enhanced with GGBS up to 20% replacement decrease progressively. Aprianti et al. [17] found that the compressive strength and the cracking potential of concrete at an early age was increased with the substitution of cement by GGBS up to 50% of the mass and suggested the appropriate amount GGBS replacement rate was not to exceed 20% of the cement mass. Silica fume is an ultrafine, amorphous powder that exhibits a pozzolanic effect. It reacts with calcium hydroxide to produce C-S-H, filling the spaces between cement particles to form a dense concrete matrix and an interfacial transition zone (ITZ). As a result, it can decrease the permeability and increase the compressive strength of concrete [18,19,20]. Sadrmomtazi reported on the effects of using silica fume as a cement replacement (at 5% and 10% of the mass) on the properties of concrete. Due to the high early reactivity and the filling effect of silica fume, the concrete has a strong affinity in terms of strength at the earliest stage (7 days) [21], and both the capillary and gel pore volumes of the mixtures decreased. The addition of silica fume was seen to have a minor effect on the pore size distribution of the ternary mixtures according to MIP tests, and it led to a significant reduction in the concrete’s permeability to chloride ions. Morphology studies and bond quality analyses using SEM revealed that the use of silica fume contributes largely to the formation of dense structures and smooth ITZs. Furthermore, the C-S-H gel structure became denser and the amount of calcium hydroxide was reduced [22]. Numerous researchers have leveraged a combination of silica fume and Ground Granulated Blast-Furnace Slag (GGBS) as an approach to augment the hydration-hardening gel material. Xu et al. [23] observed that an elevated content of GGBS led to a decrease in the compressive strength of the cement paste. However, the addition of silica fume observably amplified the compressive strength of the blended cement paste, and the compressive strength increased with the increase in silica fume content. Zhang et al. [24] found that silica fume at 2% of cement mass produced a 3% reduction in the total cumulative porosity of the cement paste. Furthermore, this addition of silica fume incurred an equivalent drop in total cumulative porosity of the cement paste. The 2% silica fume led to reduced large capillary porosity as well as medium capillary porosity when concerning high-dosage GGBS concrete.

The permeability of concrete is intrinsically tied to its overall durability and is governed by the network of pores within the hardened cement paste [25]. Hence, there is a strong correlation between a concrete’s permeability and its microstructure. Research by Sinsiri T. et al. [26] explored the impact of the fineness and shape of fly ash (FA) on concrete, revealing a decrease in slurry permeability concurrent with a reduction in porosity. Chen J. J. et al. [27] conducted tests on the strength, permeability, and porosity of several concrete types, which varied in their water–cement ratios and limestone fines content; their results showed that the addition of limestone filled the voids in the concrete, consequently lowering its porosity and leading to a decrease in both permeability and absorption. A study by Pei Y et al. [28] analyzed the connection between the permeability and porosity of cementitious materials when subjected to high-temperature heating, which indicated that a relative porosity increase of nearly 50% caused the gas permeability to elevate by two to three orders of magnitude. Sakai Y et al. [29] investigated the correlation between permeability coefficients and key pore structure indicators, including overall pore volume, critical pore size, threshold pore size, and median pore size. Their findings unveiled a robust correlation between the median and new threshold pore sizes with concrete permeability, further proposing their use as metrics for the evaluation of concrete pore structures. Additional studies by Tsivilis S et al. [30] and Zhang X et al. [31] show that average and median pore sizes, respectively, have a significant impact on the permeability and absorption of concrete. Zhang J et al. [32] highlighted that, with an increasing median and critical pore size, the intrinsic gas permeability of concrete also elevates; this trend showcases a strong correlation and the median pore size shows a slightly stronger correlation than the critical pore size. Alongside gas permeability, the transport of water within concrete can also act as a marker for material durability. Given that concrete is a hydrophilic material, it contains pores often used by various corrosive ions as channels to infiltrate the material. As a result, the capillary water absorption capacity of concrete has become one of the most critical indicators when assessing its overall durability [33].

In order to study the relationship between pore structure and transport properties, Nuclear Magnetic Resonance (NMR) and Mercury Intrusion Porosimetry (MIP) are used to measure the structure, size, and distribution of pores of different replacement SF and GGBS blended materials. The aim of this study is to investigate the effects of the combined usage of GGBS and SF on the transport properties of concrete. For the transport properties of concrete, most scholars have studied the liquid permeation and chloride ion permeation properties of concrete, and there are fewer studies on the gas permeation properties; therefore, the purpose of this paper is to study the gas transport properties of concrete and to investigate the relationship between pore structure and gas transport properties as well as capillary absorption properties.

## 2. Materials and Methods

### 2.1. Raw Material

The raw materials include Grade 52.5 ordinary Portland cement, silica fume (SF), slag (GGBS), fine aggregate, coarse aggregate, water, and superplasticizer. Mineral admixtures include SF and GGBS. Cement is produced by Qingyun Kangjing Building Materials Limited Liability Company, Dezhou, China. We used 96-grade SF with a fineness of 3000 mesh and S105-grade GGBS with a fineness of 600 mesh. SF is provided by Henan Yixiang New Material Company, Gongyi, China. GGBS is provided by Longze New Materials, Qingdao, China. River sand with an apparent density of 2.72 g/cm^3^, a bulk density of 1.60 g/cm^3^, and modulus of fineness of 2.3 were used as fine aggregate. The coarse aggregate was crushed limestone with a maximum diameter of 20 mm and an apparent density of 2.70 g/cm^3^; the size distributions of the coarse and fine aggregates are listed in Table 1. The water absorption of coarse aggregate is 2.74% and that of fine aggregate is 1.96%. The polycarboxylic-acid-based superplasticizer had a density of 1.10 g/mL, water reduction rate of over 35%, it is manufactured by Jiangsu Subot, Nanjing, China; the water used was tap water. The chemical composition and physical properties of cement and mineral admixtures are shown in Table 2; among them, C is cement. Among them, the density and specific surface area of the material are determined by GB50204-2015 [34] and GB/T 8074-2008 [35].

### 2.2. Sample Preparation

Concrete mixture sampling and specimen preparation are in accordance with the national standard GB/T 50080-2016 [36]; the sample preparation process is as follows:

(1) Weighing: weighing the raw materials according to the mix proportion in Table 3, the percentage of gel material was determined based on the cement’s mass and the W/B is the ratio of the mass of water and gel material. A, B, and C are the corresponding numbers for the three concrete comparisons in the table.
(2) Mixing: firstly, the cement, aggregate, and mineral admixture was poured into the mixer in turn, dragged to mix for 3 min, and then 1/2 of the water was added and mixed for 3 min; finally, we poured in the remaining 1/2 of the water and water reducing agent and mixed. The mixture was then be discharged after it had good fluidity. The mixer used in this study is a forced single-axis mixer with a speed of 45 rpm, which conforms to the national standard GB/T 10171-2016 [37].
(3) Forming: wipe the molds required for subsequent performance tests clean and then evenly apply a layer of release agent to each surface inside. The use of molds was in line with the construction industry standard JG237-2008 [38]. The evenly mixed concrete mixture was poured into the mold, placed on a vibrating table for compaction, and the surface of the concrete was screeded. The surface was smoothed and then covered with a layer of plastic film to prevent the loss of free water. The sample required for this experiment is 150 mm × 150 mm × 150 mm cube.
(4) Demolding and curing: the samples were demolded approximately 24 h after casting and then cured in the water at a temperature of 20 ± 2 °C
(5) After the samples were cured to the specified age, we cut the 150 mm × 150 mm × 150 mm cubes into Φ 50 × 100 mm cylinders for subsequent tests.

### 2.3. Experimental Methods

#### 2.3.1. Microscopic Tests

(1) Nuclear Magnetic Resonance (NMR) test

In this study, the MicroM12-025VR NMR core analyzer was used for the LF-NMR relaxometry test. It is manufactured by Shanghai Newmax Electronic Technology Co., Ltd, Shanghai, China. The instrument had a constant magnetic field of 0.5 T and a radio-frequency coil of 60 mm in diameter, operating at 23.4033 MHz and a magnet temperature of 32 °C. The test sample is a cube with a side length of 10 mm. Before the LF-NMR test, the samples were re-saturated using high-pressure saturator for 48 h. To prevent the effect of water evaporation on the test, the samples were wiped with paper towels after removal from the saturator and sealed with plastic wraps.

The LF-NMR can analyze the interaction between the magnetic field and spin of hydrogen protons and produce signals called relaxation time. The relaxation time comprises longitudinal relaxation time (*T*_1_) and transverse relaxation times (*T*_2_). Since the longitudinal relaxation time (bulk relaxation time) is much greater than the transverse relaxation time (surface relaxation time), the *T*_1_ can be neglected. The larger the pore radius is, the longer the *T*_2_ relaxation time is [39,40]. According to the principle of NMR, the relationship between *T*_2_ relaxation time and the specific surface area of pores can be determined using the following equation:

(1) $$\frac{1}{T_{2}}=\rho \frac{A}{V}$$

where ρ is the relaxation strength, which is related to the material category; *A* is the pore surface area of the sample; and *V* is the total pore volume of the sample.

(2) Mercury Intrusion Porosimetry (MIP) test

In this study, the porosity and microstructure characteristics of concrete were investigated using a AUTOPORE 9500 Mercury Porosimeter. This instrument is manufactured by McMurray Tick Instruments Ltd., Shanghai, China. A cube with a side length of 10 mm was cut from the concrete sample and contained both the mortar and aggregate. In order to reduce the influence of moisture in the samples on the test results [41,42], before testing, we placed the samples in an oven at 105 ± 5 °C for 2 h.

#### 2.3.2. Permeability Test

(1) Gas permeability test

**Figure 1:**
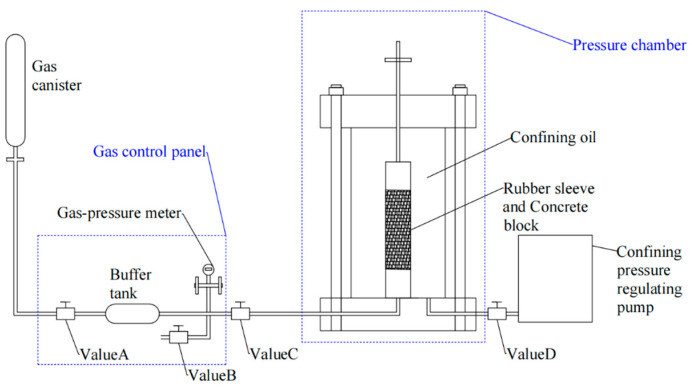
Schematic diagram of gas permeability test.

The experiment was tested using the gas penetration standard RILEM116-PCD [43]. The instrument of gas permeability consists of a gas transmission control system, a confining pressure chamber, and a high-precision, servo-confining, pressure-loading system. The experiment uses inert gas argon as the permeation medium, and the schematic diagram is shown in Figure 1.

Cylindrical concrete specimens of Φ 50 × 100 mm were placed in the confining pressure chamber and tightened using a jacket and a hose clamp before the test. Nine gas permeability tests were conducted for each proportion of the concrete specimens at inlet pressures of 5, 10, and 15 bar and confining pressures of 3, 5, and 10 MPa. Gas permeability was measured using a one-dimensional steady-state flow method based on Darcy’s law, which can be expressed as follows:

(2) $$V_{x}=-\frac{K_{x}}{\mu }\frac{dP(x)}{dx}$$

where *K_x_* represents gas permeability, *V_x_* is the gas flow rate, *μ* is the gas viscosity coefficient, and *P*(*x*) is the function of the pressure inside the sample.

The upstream is connected to a gas storage tank to maintain a stable inlet pressure through the gas transmission control system, and the outlet is the atmospheric pressure. As the test proceeds, the gas flow goes inside the sample; the initial pressure value *P*_1_ of the buffer gas tank in the control system decreased by ∆*P*_1_ at time ∆*t*. Assuming that the gas flow is stable during ∆*t*, noting that Pm is the mean gas pressure of the buffer gas tank, *P_m_* = *P*_1_ − (*P*_1_/2). According to the rational gas law, the mean flow rate of the specimen during time ∆*t* can be expressed as

(3) $$\bar{Q}=\frac{\Delta P_{1}V_{1}}{P_{m}\Delta t}.$$

The calculation formula for gas permeability is as follows:

(4) $$K=\frac{2\mu h∆P_{1}V_{1}}{S\left(P_{m}^{2}-P_{o}^{2}\right)}$$

where *K* is the gas permeability coefficient, *h* is the height of the sample, *S* is the area of the sample, *P_o_* is the atmospheric pressure, and *V*_1_ is the buffer gas tank volume.

(2) Capillary water absorption test

**Figure 2:**
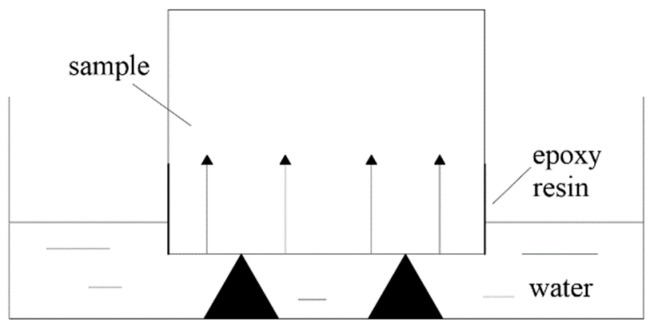
Schematic of capillary water absorption test.

The experiment was tested using the ASTM-C1585-13RILEM116-PCD [44] hydration standard. The well-cured cylindrical sample was put into the oven at 65 °C and dried to a constant weight, and then naturally cooled to room temperature. The sample was sealed using epoxy resin around the sample to ensure that the capillary water absorption was carried out along the one-dimensional direction. After that, the sample was placed on the bracket in the sink, avoiding the bottom of the test block from directly touching the sink, and water was added to the sink so that the water did not go over the bottom of the sample by 1~2 mm. The schematic of the capillary water absorption test device is shown in Figure 2.

The cumulative water absorption of the sample can be obtained from Equation (5). The test results are taken as the average of three test samples.

(5) $$i=\frac{∆W}{A\rho_{w}}$$

where *i* is the cumulative water absorption per unit area, mm; Δ*W* is the water absorption, g; *A* is the area of contact between the test sample and water, cm^2^; and $\rho_{w}$ is the density of water, 1 g/cm^3^;

The cumulative water absorption of concrete is related to the square root of time, as follows:

(6) $$i=a+k\times t^{0.5}$$

where *k* is the water absorption rate, mm/min^1/2^; *a* is the surge in water absorption, mm; and *t* is the time of capillary water absorption, min.

## 3. Results and Analysis

### 3.1. Pore Structure

#### 3.1.1. NMR Test

**Figure 3:**
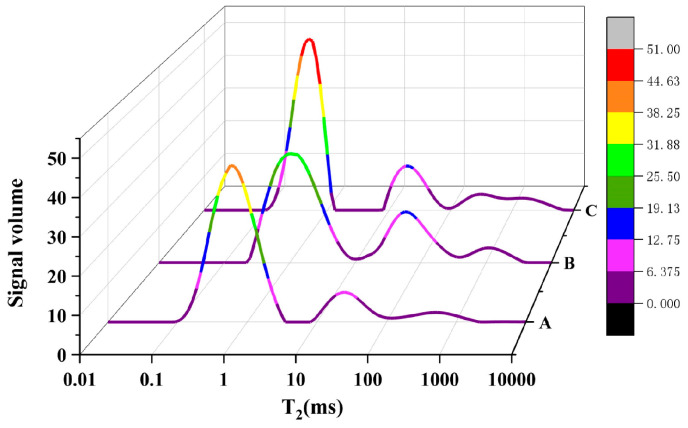
T2 spectrum of test concrete obtained using the NMR method.

The Nuclear Magnetic Resonance (NMR) test can generate an accurate estimate of the gel pores (*T*_2_ ≤ 2.5 ms), capillary pores (2.5 ms ≤ *T*_2_ ≤ 50 ms), and stomatal openings (*T*_2_ > 100 ms) by analyzing the total relaxation time, *T_2_*, spectrum. As demonstrated in Figure 3, the obtained *T*_2_ signal can be represented as a *T*_2_ spectrum, which shows each concrete proportion having primary and two to three subsidiary *T*_2_ spectral peaks, primarily located within the gel pores [32,45]. The explicit difference between the primary peak and the first secondary peak in samples A and C indicates the absence of a direct channel between gel pores and capillary pores, suggesting these samples possess high denseness without pore throat formation [46]. The pour space within pores A and C in the concrete is less developed than the adsorption space, resulting in low interconnection, subsequently hindering gas transportation. The *T*_2_ spectra indicate that, in terms of the primary peak appearance time, the peaks for concrete samples A and C appear earlier than those for sample B and are higher. According to NMR principles, the entire area under each distribution curve, or the total porosity, represents the total pore water content within the concrete. Consequently, the size connection of porosity among various concrete samples with mineral admixtures can be determined by analyzing the *T*_2_ spectral area. Concrete A and C exhibit a somewhat larger peak area in the primary peak region, suggesting a higher number of micro-pores. This is attributable to the fact that silica fume possesses lesser fineness than Ground Granulated Blast-Furnace Slag, which aids in filling the cementitious material space, thus enhancing the concrete’s microstructure by reducing pore size.

**Figure 4:**
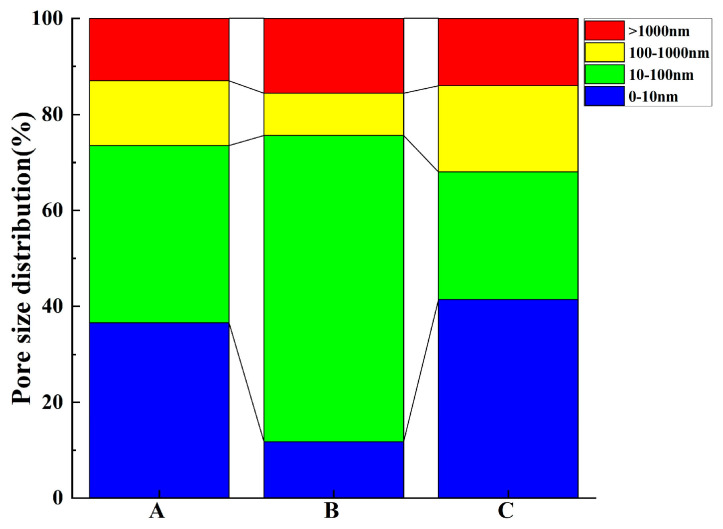
Pore size distribution of test concrete using the NMR method.

Four categories of concrete pore sizes were delineated, namely, gel pores (d < 10 nm), micro-capillary pores (10 < d < 100 nm), macro-capillary pores (100 < d < 1000 nm), and fractures (d > 1000 nm) [3,47]. As illustrated by the distribution of pore size proportions in Figure 4, the macro-capillary pores and fractures make up a comparatively negligible percentage of the total pores in all three concrete samples. In sample B, micro-capillary pores predominate, trailed by fractures and gel pores; in sample A, gel and micro-capillary pores amount to nearly identical proportions, predominating the sample. In sample C, the gel pores are the most abundant, with micro-capillary pores being the least prevalent.

By analyzing the *T*_2_ spectrum and pore size distributions, it is feasible to define the pore size parameters illustrated in Table 4. Here, the median pore size is defined as the pore size that equates to a 50% signal intensity in the NMR test.

#### 3.1.2. MIP Test

**Figure 5:**
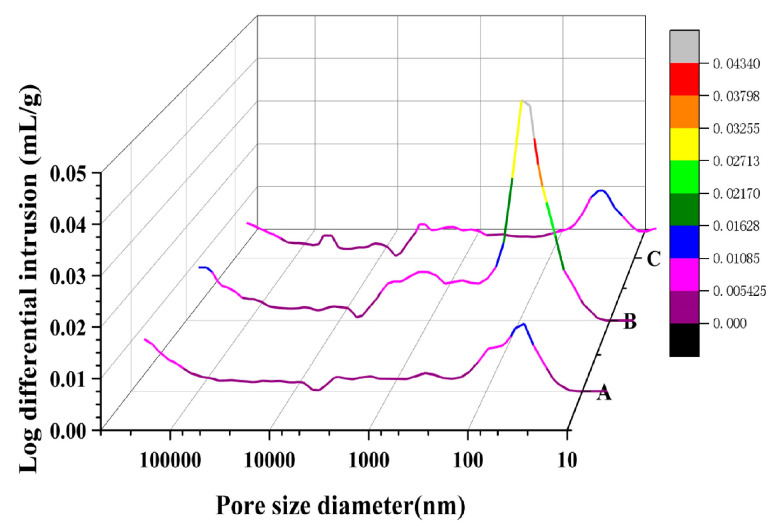
Mercury injection diagram of test concrete obtained using the MIP method.

The pore size distributions of concrete proportions A, B, and C, as elucidated using the MIP method, are displayed in Figure 5. The sample mixed solely with SF, sample A, possesses the smallest total pore volume at 0.018 mL/g. In contrast, sample B, mixed uniquely with GGBS, exhibits the largest total volume at 0.043 mL/g—nearly twice the volume of sample A. Inconsistencies arising from the variances in test concepts and test ranges manifest in results from NMR and MIP testing: MIP analysis suggests a higher porosity for three samples than MIP results. The intrinsic “ink bottle effect” ascribed to MIP [48] confines its pore measurement range to sizes larger than 5 nm, encompassing a range of 5–350,000 nm. Conversely, NMR embraces a more vast range, from 2 to 20,000,000 nm. So, NMR measurements provide more complete and representative data. However, the NMR results may be somewhat impacted by the presence of metallic elements in the concrete material, resulting in measured values that are lower than actual values.

**Figure 6:**
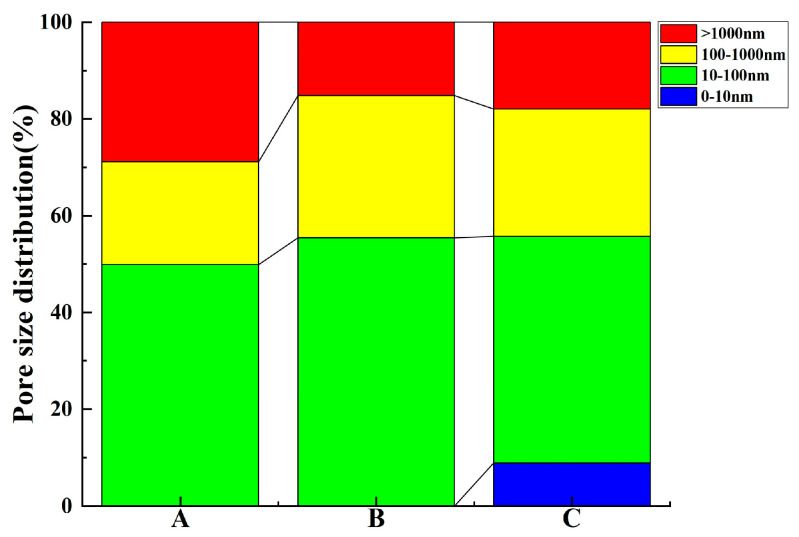
Pore size distribution of test concrete using the MIP method.

The divergence in pore size distribution is especially prominent for samples A and B within the ranges 10–1000 nm, more than 1000 nm, and notably in zones with fractures larger than 1000 nm, as visible from the percentage distribution in Figure 6. Importantly, sample A displays a markedly higher percentage of fractures compared to sample B. The spherical particles and smooth surface of GGBS foster cement particle dispersion, thus enhancing concrete fluidity. Conversely, although SF possesses a larger surface area, it demands a higher water content. Substituting SF for cement results in an augmentation in water requirement and a reduction in fluidity, yielding a rough and pitted surface, culminating in a “honeycomb” effect, and inducing a higher incidence of cracks. Thus, while GGBS addition accentuates concrete fluidity and reduces macro-capillary pores and crack porosity, the integration of SF effectively obliterates cement pores, refines pore size, and improves the microstructure, albeit at the expense of concrete fluidity, and contributes to an increase in macro-capillary pores and crack porosity.

From Figure 5, the critical pore size and most probable pore size can be extrapolated; these values are tabulated in Table 5. The ‘critical pore size’ refers to the pore size necessary for the establishment of a connecting channel spanning from one side of the sample to the other [49]. Significantly, the inflection point on the cumulative pore size distribution curve aligns mathematically with the critical pore size [29,50]. On the other hand, the ‘most probable pore size’ is associated with the pore size that corresponds to the peak value on the differential distribution curve of pore sizes, as determined using piezometric mercury intrusion.

### 3.2. Transport Properties

#### 3.2.1. Gas Permeability

Nine apparent gas permeability values for three concrete mixtures (A, B, and C) were investigated at confining pressures of 3, 5, and 10 MPa and at input pressures of 5, 10, and 15 bar, respectively, as Table 6 illustrates.

(1) Apparent gas permeability

**Figure 7:**
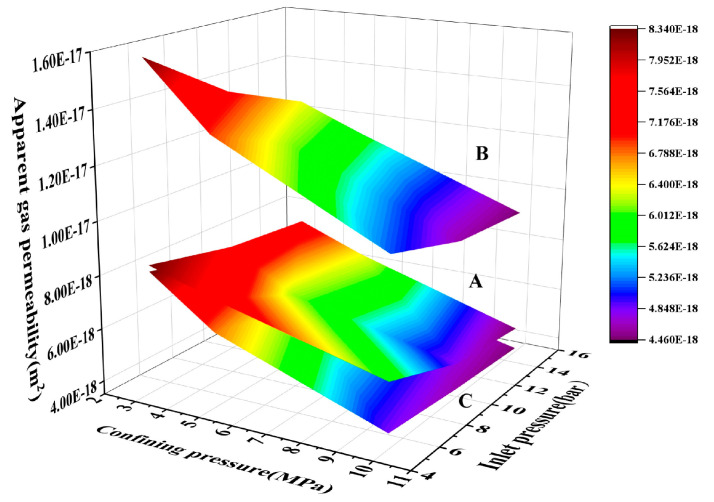
The variation law of apparent gas permeability under the coupling effect of confining pressure and inlet pressure.

Figure 7 shows the progression of apparent gas permeability for various concrete sample proportions under different confining and inlet pressure couplets. Figure 7 indicates specific characteristics of the permeability of the concrete samples, irrespective of their precise proportions. The gas permeability of the concrete tends to decrease progressively with increasing confining pressure. Additionally, as the inlet gas pressure increases, the gas permeability initially decreases, subsequently followed by an increase. Under the same confining and inlet pressure, the permeability coefficient is ranked sequentially as C < A < B. Moreover, permeability appears to be more influenced by variations in confining pressure than in inlet pressure.

**Figure 8:**
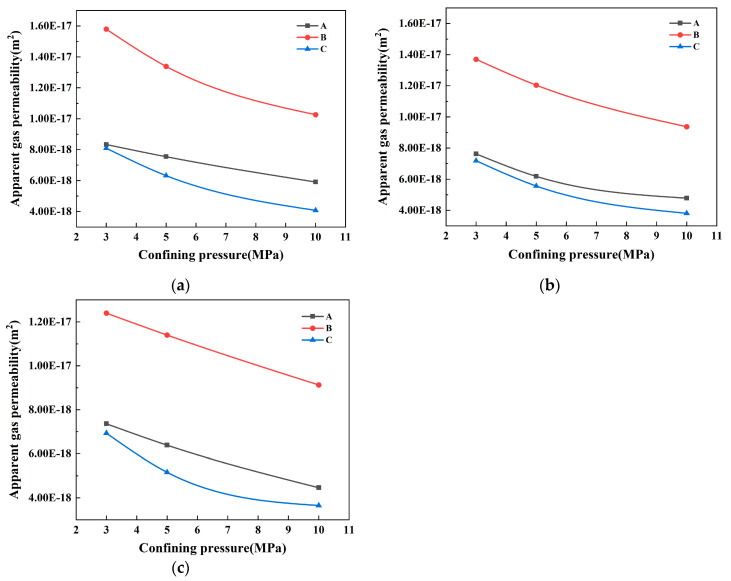
The variation law of *K_app_* with confining pressure under different inlet pressure conditions: (**a**) inlet pressure 5 bar; (**b**) inlet pressure 10 bar; (**c**) inlet pressure 15 bar.

Figure 8 shows the apparent permeability of concrete samples at various inlet pressures, plotted against the confining pressure, utilizing data from Table 6. It becomes clear that the apparent gas permeability values of the three concrete proportions, A, B, and C, decrease steadily as the confining pressure increases, independent of the level of inlet pressure. Furthermore, the rate at which this reduction takes place also discontinue as confining pressure rises.

This study was undertaken to better understand the effects of confining pressure on the pore structure of the concrete. Within lower ranges of confining pressure, enhancing this pressure effectively improves the pore structure of the concrete sample. Heightened confining pressure can cause both large and small pores within the sample to become constricted and sealed off. As a result, seepage channels within the sample undergo progressive compaction, increasing the resistance of the concrete to permeability. Moreover, as the confining pressure increases further, the ability of these inner seepage channels to be compressed declines, as does the degree of compression experienced by them. As such, the change in permeability gradually slows with increases in confining pressure.

(2) Intrinsic gas permeability

**Figure 9:**
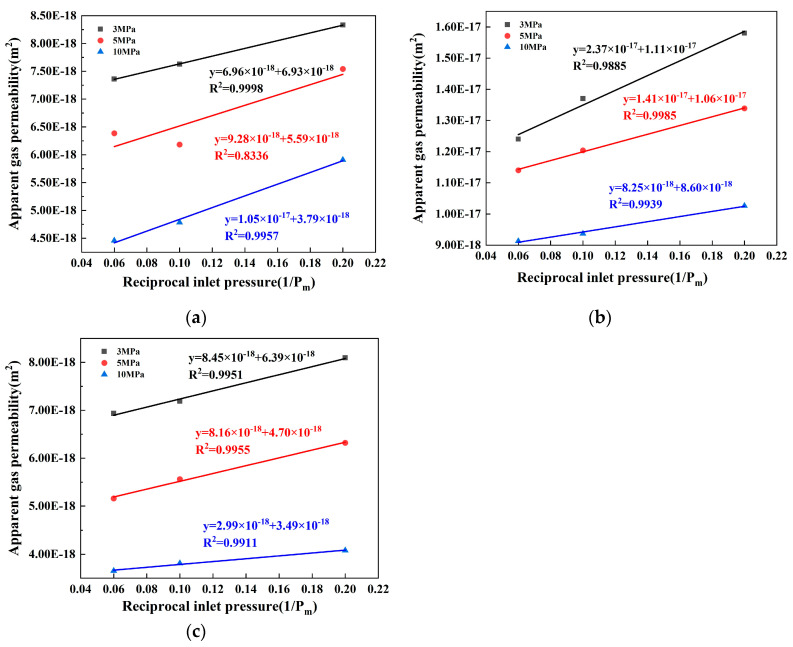
Fitting diagram of the relationship between the reciprocal inlet pressure and apparent gas permeability of concrete with different proportions: (**a**) concrete of A; (**b**) concrete of B; (**c**) concrete of C.

The data in Table 6 are used to establish and fit the relationship between the apparent gas permeability of concrete samples and the reciprocal of inlet pressure under various confining pressure conditions. This correlation is clearly demonstrated in Figure 9a–c. The plots show a strong correlation between the reciprocal of the inlet pressure and the apparent gas permeability. After fitting, the coefficient of determination between them is almost above 0.9, indicating a significant relationship between these two variables.

In order to accurately reflect the true permeability coefficient and the actual condition of the pores within the concrete, the intrinsic gas permeability (*K_int_*) has been proposed [51,52]. This is a widely used overseas approach that accounts for changes in the gas transmission mechanism and gas permeability coefficient based on the test pressure. Hence, before a correlation between gas permeability and the structure of the porosity can be examined, tests for gas permeability should be conducted under a variety of pressures to neutralize the influence of pressure.

Equation (7) has been utilized to make corrections to the intrinsic gas permeability of the concrete samples, as derived from Figure 9a–c. These corrections are made in line with the Klinkenberg effect, with the corrected results outlined in Table 7:

(7) $$K_{app}=K_{int}\left(1+\frac{\beta }{P_{m}}\right)$$

where *K_app_* is the apparent gas permeability, *K_int_* is the intrinsic gas permeability, *β* is the Klinkenberg factor, and *P_m_* is the average gas pressure at the inlet end.

When combining the data in Table 6 and Table 7, it can be observed that the apparent gas permeability coefficient is consistently higher than the intrinsic gas permeability coefficient, regardless of the confining pressure applied. Both coefficients follow the same pattern: the compound mixture of SF and GGBS in the concrete C and the single mixed SF in the A type concrete present greater resistance to gas transmission than the single mixed GGBS in the concrete B and the compound mix of SF and GGBS in concrete C. This outcome is mainly due to the extremely small particle size of the SF. The addition of SF enhances the hydration process of cement, leading to the refinement of hydration products of calcium hydroxide. Consequently, a C-S-H (calcium silicate hydrate) gel of high strength and density is formed, which is more efficient in resisting transmission of media by reducing the number of connecting channels and limiting water and gas passage through the concrete. Moreover, the combined usage of SF and GGBS can potentially induce a ‘Microaggregate Effect’, where the particle sizes of SF and GGBS form a gradient with cement particles and fit with one another. This can result in further reductions in spaces between fine aggregate particles, augmenting the concrete’s density and subsequently, enhancing its permeability.

#### 3.2.2. Capillary Water Absorption

**Figure 10:**
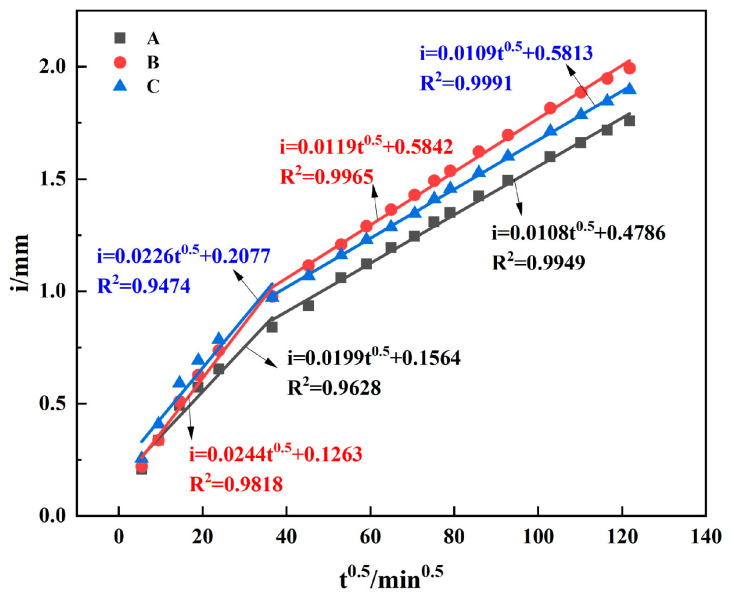
Capillary water absorption.

The quantity and distribution of pores within a material, demonstrated by concrete’s surface tension and pore absorption, are primarily responsible for capillary absorption. Capillary absorption, which is often used to evaluate concrete’s durability, pronounces the absorption and movement of water through the capillaries of porous substances. Consequently, it can directly symbolize the capillary pore structure of concrete’s surface layer. Figure 10 illustrates the dispersion of capillary water absorption data points for various concrete mixes over a period of 10 days. The water absorption for each concrete mix reveals two distinct stages, which increase with the square root of the water absorption time. Initially (*t* ≤ 570 min), concrete’s capillary water absorption coefficient is high and the water absorption rate is fast. After this initial stage, however, the capillary water absorption coefficient significantly decreases, and the rate at which water is absorbed slows down. Table 8 presents the coefficient of determination following a fitting to *t*^1/2^, along with the two stages’ capillary absorption coefficient. The table displays that, in both the initial and later stages of water absorption, the water absorption coefficients for concrete types A and C are lower than that of type B. This suggests that A and C concrete types absorb less water than B concrete, while A concrete, with a single addition of SF, continually shows a better ability to resist water intrusion than C concrete, which has a double addition of SF and GGBS. However, with respect to resisting gas permeation—as highlighted in Section 3.2.1—the C concrete type presents a better performance than the A type. This analogy follows the same pattern as the gas permeability coefficient for the three concrete proportions in Section 3.2.1. The concrete’s permeability performance and the porosity determined via NMR analysis demonstrate that a higher porosity does not necessarily mean a reduced ability to withstand media transport; hence, porosity alone cannot serve as an accurate representation of concrete’s permeability.

### 3.3. Relationship between Pore Structure and Permeability

#### 3.3.1. Relationship between Porosity and Permeability

**Figure 11:**
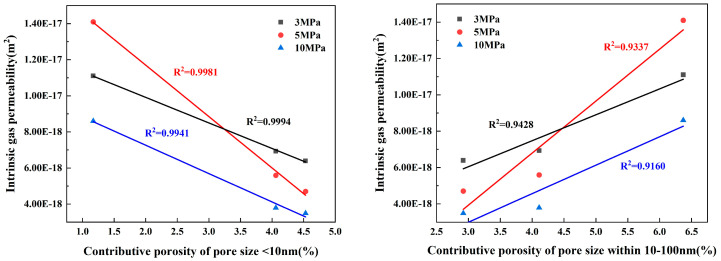

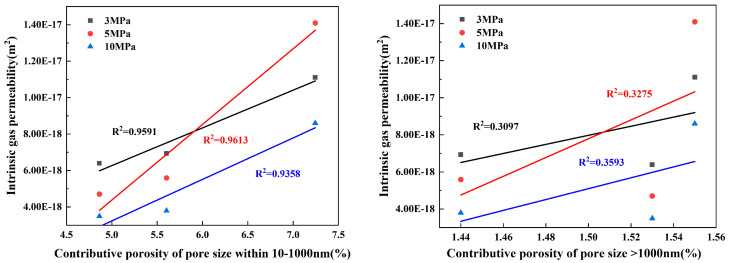
Relationship between intrinsic gas permeability coefficient and contributive porosity.

**Figure 12:**
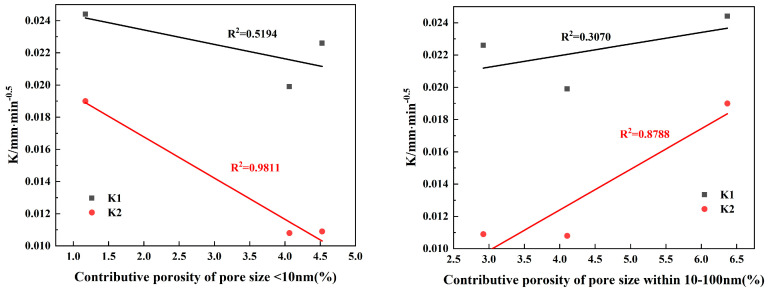

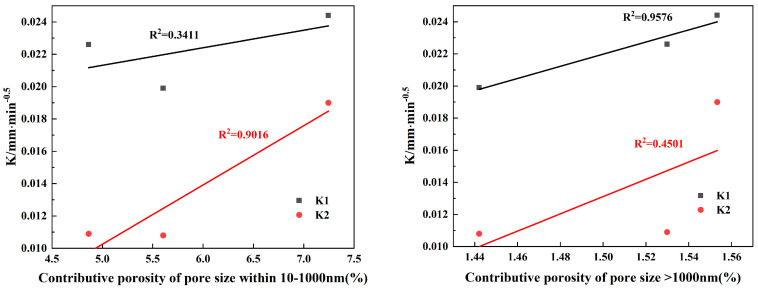
Relationship between capillary water absorption coefficient and contributive porosity.

The manner in which media of differing sizes pass through pores can significantly vary, necessitating a thorough analysis of how pore size can impact permeability. As a response, this paper suggests an indicator known as ‘contributing porosity’. Contributing porosity [47] combines the impacts of pore size and porosity on water and gas permeability. It is the product of the porosity and the percentage of a specific pore size within the total porosity, representing the ratio of the pore volume within a certain pore size range to the whole sample volume. Utilizing the data on contributing porosity and the capillary water absorption coefficients from Table 4, along with the gas permeability coefficients at varying confining pressures, fitting relationship charts are created, which are displayed in Figure 11 and Figure 12. These figures show the fitting relationship between the intrinsic gas permeability at various confining pressures and capillary water absorption and the contributing porosities of different pore size ranges, as determined via NMR analysis.

Figure 11, with extremely high coefficient of determinations (R^2^ > 0.99), demonstrates a significantly negative correlation between the contributing porosity of pores within the <10 nm pore size range and gas permeability at all three given confining pressures. This indicates that having an increase in the quantity of pores within the <10 nm range is beneficial for reducing the gas permeability of concrete. Contrarily, when the pore size lies between 10 and 1000 nm (encompassing micro- and macro-capillary pores), the contributing porosity has a prominent positive correlation with the intrinsic permeability, with coefficient of determinations of 0.9 or above (R^2^ > 0.9). Moreover, this correlation tends to be progressively stronger when the pore size range broadens from 10–100 nm to 10–1000 nm. This suggests that an enhancement in the concrete’s pore size proportion within the 10–1000 nm range facilitates the movement of gas molecules within the material. In other words, an increase in the proportion of capillary pores may lead to the formation of more interconnected pores within the concrete, consequently augmenting the material’s gas permeability. However, for pores larger than 1000 nm, the correlation between contributing porosity and gas permeability is somewhat weak, with a coefficient of determination of just 0.3.

Figure 12 reveals that an increase in pores smaller than 10 nm negatively affects the late stage of water absorption in concrete. The contributing porosity for pores in the size range <10 nm shows a highly negative correlation with the late capillary water absorption coefficient (R^2^ = 0.9811). However, its correlation with initial primary capillary water absorption is low. This pattern is consistent with findings reported by [53]. As the pore size range expands from 10–100 nm to 10–1000 nm, the contributing porosities show substantially strong associations with late water absorption. The correlation strength with water absorption performance also increases. This suggests that capillary pores between 10 and 1000 nm are primarily influential in the late stage of capillary water absorption. Furthermore, there is a strong correlation between the initial water absorption and the late-stage absorption coefficient. However, the correlation between the contributing porosity of pores with size ranges above 1000 nm (stomatal) is weak. This observation aligns with the findings of Hou et al. [54], who reported that pores larger than 1000 nm might also contribute to transport to a smaller extent.

By comparing Figure 11 and Figure 12, it becomes clear that the correlations between gas permeability and contributing porosity are generally stronger than those between capillary water absorption and contributing porosity, particularly in the <10 nm, 10–100 nm, and 10–1000 nm pore size ranges. Gas permeability coefficient of determinations are above 0.9, while some capillary water absorption coefficient of determinations are below 0.9. The Klinkenberg effect [55] can provide an explanation for this discrepancy. According to this effect, when the pore size is comparable to the mean free path of gas molecules, there could be gas “slip” at the pore wall, increasing the rate of gas flow. On the other hand, when water enters capillary pores via capillary absorption—primarily facilitated by concrete surface tension and pore absorption—it is likely to be hampered by friction against the side walls. Argon molecules leveraged for permeability assessments have a rather small dynamic size and can traverse the pore structures with relative ease.

#### 3.3.2. Relationship between Pore Size and Permeability

**Figure 13:**
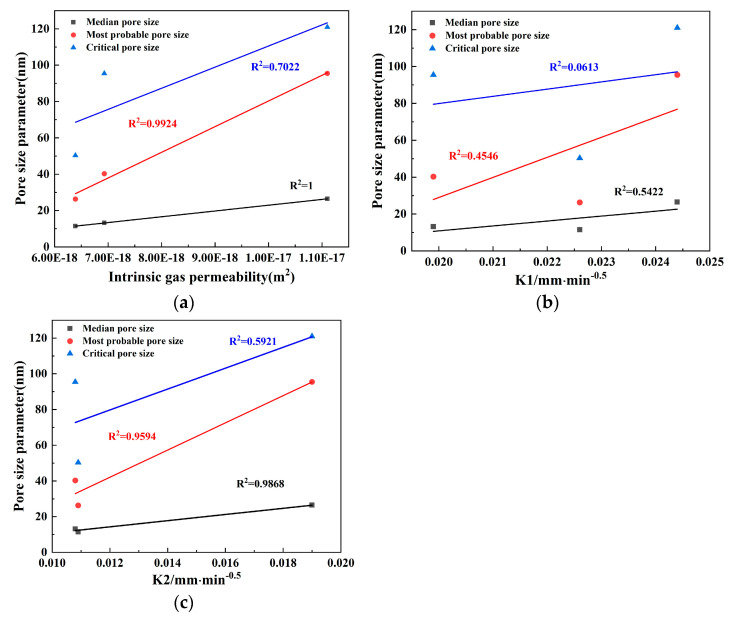
The relationship between pore size parameters and permeability.

The analysis in this section focuses solely on the relationship between intrinsic gas permeability and pore size parameters at a confining pressure of 3 MPa as the rules exhibited by the intrinsic gas permeability of concrete are consistent across differing confining pressures. All instances share the same relationship with the contributing porosity. Figure 13 provides a visual representation of the relationships in question, which include the fitted plots of the intrinsic gas permeability coefficient, the initial capillary absorption coefficient, and the late absorption coefficient against the median, critical, mean, and most probable pore sizes, respectively, each at a confining pressure of 3 MPa. These are delineated in Figure 13a–c.

As illustrated in Figure 13a, there is a compelling positive correlation between the intrinsic gas permeability and the median pore size, with an impeccable coefficient of determination of one (R^2^ = 1). Additionally, a strong positive correlation can be observed between the intrinsic permeability and the most probable pore size, dictating a coefficient of determination of 0.9924 (R^2^ = 0.9924). It is worth noting that, according to MIP technology, the mean pore size aligns with the most probable pore size as the pore openings are based on a normal distribution. The graph also reflects a strong positive correlation between the mean pore size and permeability, boasting correlation values exceeding 0.97. Consequently, the mean pore size can serve as an appropriate parameter to describe the permeability of concrete. However, a mere coefficient of determination of 0.7 highlights a weaker link between critical pore size and permeability. The mean free path of the test gas molecules (argon, in this case) aligns more seamlessly with the median pore size than with the critical pore size, causing the median pore size to exert a more profound impact on the gas permeability characteristics [56].

These findings, thus, lead to the concession that, as proxies for permeability in terms of pore structure, the median and most probable pore sizes are far more powerful indicators than the critical pore size [29]. It is also indicated that the median pore size can be determined independently, without examiner intervention, and it demonstrates the best coefficient of determination. Conversely, according to Figure 13b, the water absorption coefficient during the initial capillary water absorption phase of concrete shows an insignificant correlation with the critical pore size and a weak correlation with the median and most probable pore sizes. This demonstrates that the initial capillary water absorption cannot be accurately depicted by either the median, the most probable, or the critical pore sizes. However, in the late stage of water absorption, as showcased in Figure 13c, the water absorption coefficient exhibits a strong positive correlation with the median and most probable pore sizes (mean pore size), with correlation values surpassing 0.95. Consequently, a more precise description of the late capillary water absorption coefficient can be achieved by leveraging the median and most probable pore sizes.

When correlating coefficients of each pore size parameter with gas permeability and capillary absorption, respectively, a higher correlation between gas permeability and the most probable and median pore sizes was observed compared to that of capillary absorption. This suggests that gas permeability is noticeably more sensitive to changes in pore structure than capillary absorption in all three types of concrete.

## 4. Conclusions

The purpose in this study was to investigate the effect of GGBS and SF dosages on concrete. The pore structure and transport properties were analyzed through experiments. The following conclusions can be obtained:

1. Based on the NMR and MIP test results, the incorporation of SF and GGBS significantly reduced the most probable pore size and the content of harmful pores in concrete. When incorporated at lower substitution rates, silica fume had a more pronounced impact on the pore structure of concrete materials than Ground Granulated Blast-Furnace Slag. It is clear that different combinations of mineral admixtures can produce concretes with better performance, and the selection of these combinations should be based on the physical properties associated with the expected performance of the concrete.
2. GGBS replacement without SF had no significant effect on the transport properties. To reduce the permeability, the incorporation of SF in concrete is very effective. In binary systems, adding SF refined the pore structure and improved the transport properties more than GGBS by decreasing the proportion of capillary pores, even with only a 15% addition. However, in ternary systems, SF and GGBS synergistically did not show a significant effect on the permeability. Furthermore, the experimental findings strongly suggest that both the median and most probable pore sizes in concrete significantly impact its transport properties.
3. Distinct differences exist in the porosity and pore size distribution, as determined by Mercury Intrusion Porosimetry and Nuclear Magnetic Resonance tests. It was observed that NMR proves valuable in ascertaining microscopic porosity, while MIP exhibits proficiency in identifying macro-porosity. However, variations in specimen size and sample pretreatment introduced a degree of randomness into the MIP measurements. NMR test outcomes are potentially more representative of the real porosity of concrete, but it is also critical to understand how metallic components in concrete materials affect things.
4. The results of contributing porosity for different pore sizes show the following: An increase in <10 nm pores has a positive effect on reducing the gas permeability coefficient and the capillary late absorption coefficient of concrete. In the ranges of 10–100 nm and 10–1000 nm, the gas permeability coefficient and the capillary late absorption coefficient both increase with the increase in contributing porosity, which show highly correlated positive correlations, but the correlation between gas permeability and contributing porosity correlates better than capillary water absorption. Pores > 1000 nm correlate poorly with gas permeability but show a highly correlated positive correlation with the capillary initial water absorption coefficient.

## 5. Future Research

The transport properties and pore structures of concrete materials are very complex, and the connection between the two is affected by various factors. Limited by the precision of experimental equipment and instruments, the research methods and results of this thesis have certain shortcomings; there is still space for further improvement.

In terms of microscopic studies, such as NMR and MIP microscopic tests, a larger volume of samples can be selected in future studies to more comprehensively reflect the microstructural characteristics of concrete and its relationship with transport properties, or CT tests can be conducted to obtain more complete and intuitive microscopic information.

In terms of transport properties, the transport tests include the gas permeability test, liquid permeability test and chloride permeability test, and there are more articles on chloride permeability; this paper only investigates the gas permeability properties of concrete materials, so the liquid permeability properties of concrete materials should be explored under different permeable mediums (e.g., water and ethanol) in subsequent tests.

## Figures and Tables

**Table 1:** The size distribution of coarse aggregate and fine aggregate.

Screen Size (mm)	Cumulative Sieve Residue (%)	
	Coarse Aggregate	Fine Aggregate
26.5	0	0
19.0	9.7	0
16.0	22.6	0
9.50	61.7	0
4.75	89.3	5.1
2.36	99.1	15.3
1.18	/	26.2
0.6	/	41.8
0.3	/	69.1
0.015	/	90.4
Screen tray	100.0	100.0

**Table 2:** Chemical composition and physical properties of cement and mineral admixtures.

Binders	Chemical Compositions (%)							Specific Surface Area (m^2^/kg)	Density (g/cm^3^)
	CaO	SiO_2_	Al_2_O_3_	MgO	Fe_2_O_3_	SO_3_	Cl^−^		
C	59.27	20.86	9.28	2.6	3.74	1.85	0.04	349	3.10
SF	-	96.2	-	0.80	0.13	-	0.07	20,000	2.23
GGBS	35.30	34.50	16.70	5.01	1.50	1.24	-	628	2.93

**Table 3:** Concrete mixture proportions.

Group	Binder kg/m³	Binder Composition (%)			W/B	Water kg/m³	Superplasticizer kg/m³	Coarse Aggregate kg/m³	Fine Aggregate kg/m³
		C	SF	GGBS					
A	900	85	15	0	0.25	225	18	616	923
B	900	85	0	15	0.25	225	18	616	923
C	900	85	7.5	7.5	0.25	225	18	616	923

**Table 4:** Pore size parameters of NMR.

Method		Porosity (%)	Contributive Porosity within Different Range of Pore Size in Concrete (%)				Median Pore Size (nm)
			<10 nm	10–100 nm	100–1000 nm	>1000 nm	
NMR	A	2.26	29.44	42.47	17.18	10.91	13.16
	B	3.93	11.75	63.88	8.79	15.58	26.44
	C	5.35	41.45	26.56	17.98	14.01	11.45

**Table 5:** Pore size parameters of MIP.

Method		Porosity (%)	Most Probable Pore Size (nm)	Critical Pore Size (nm)
MIP	A	4.38	40.277	95.451
	B	9.82	95.474	120.991
	C	6.45	26.292	50.348

**Table 6:** Apparent gas permeability *K_app_* (m^2^) at different confinement pressures and inlet pressures.

Group	Confinement Pressures (MPa)	Inlet Pressures (bar)		
		Pi = 5	Pi = 10	Pi = 15
A	3	8.33 × 10^−18^	7.63 × 10^−18^	7.36 × 10^−18^
	5	7.54 × 10^−18^	6.18 × 10^−18^	6.39 × 10^−18^
	10	5.91 × 10^−18^	4.78 × 10^−18^	4.46 × 10^−18^
B	3	1.58 × 10^−17^	1.37 × 10^−17^	1.24 × 10^−17^
	5	1.34 × 10^−17^	1.20 × 10^−17^	1.14 × 10^−17^
	10	1.03 × 10^−17^	9.37 × 10^−18^	9.13 × 10^−18^
C	3	8.10 × 10^−18^	7.91 × 10^−18^	6.93 × 10^−18^
	5	6.32 × 10^−18^	5.56 × 10^−18^	5.16 × 10^−18^
	10	4.08 × 10^−18^	3.81 × 10^−18^	3.65 × 10^−18^

**Table 7:** Intrinsic gas permeability *K_int_* (m^2^).

Confining Pressure (MPa)	A	B	C
3	6.93 × 10^−18^	1.11 × 10^−17^	6.39 × 10^−18^
5	5.59 × 10^−18^	1.06 × 10^−17^	4.70 × 10^−18^
10	3.79 × 10^−18^	8.60 × 10^−18^	3.49 × 10^−18^

**Table 8:** Capillary water absorption coefficient of determination.

	Initial Stage		Late Stage	
	K1	R2	K2	R2
A	0.0199	0.9628	0.0108	0.9949
B	0.0244	0.9818	0.0119	0.9965
C	0.0226	0.9474	0.0109	0.9991

## Data Availability

The data presented in this study are available on request from the corresponding author.
